# Ultrasonographic Appearances of Cervical Lymph Nodes in Healthy Turkish Adults Subpopulation: Preliminary Study

**DOI:** 10.2174/1874210601711010404

**Published:** 2017-06-30

**Authors:** Özlem Okumuş, Merve Dönmez, Filiz N. Pekiner

**Affiliations:** 1 Department of Oral Diagnosis and Radiology, Faculty of Dentistry, Istanbul Kemerburgaz University, Istanbul, Turkey; 2 Department of Oral Diagnosis and Radiology, Faculty of Dentistry, Marmara University, Istanbul, Turkey

**Keywords:** Cervical, Lymph nodes, Ultrasonography, Normal anatomy

## Abstract

**Objectives:**

The aim of the study was to assess whether there was any relation between age, gender and body mass index (BMI) and nodal forms and vascular type in healthy Turkish adults.

**Study Design:**

Three neck areas in 25 wholesome patients who were aged from 21 to 58 years, were assessed by gray-scale and color doppler ultrasonography. Ultrasonographic examinations were performed using an ALOKA Prosound Alpha 6 (Hitachi Aloka Medical Systems, Tokyo, Japan) and the images were obtained with a 7.2 MHz linear array transducer. Hajek’s categorization of cervical lymph nodes for sonographic analysis was used. The ultrasonographic characteristics like size, shape, short axis/long axis ratio (S/L), hilum were evaluated. Ultrasonographic examinations of upper cervical, submandibular and submental lymph nodes were carried out and recorded.

**Results:**

The mean age of patients was 31.84±12.80 years. The ratios of lymph nodes with avascular pattern were 96% for the upper cervical lymph area, 92% for the submandibular area and 96% for the submental area. The lowest and highest ratios of short to long axis diameter (S/L) were calculated as 0.18 and 0.66 in all areas. Most normal nodes in the study were oval with an S/L ratio of less than 0.5.

**Conclusion:**

Normal cervical lymph nodes are oval, with an unsharp border and an echogenic hilum but no relation between the age, gender and BMI. Also ultrasonography is an applicable imaging modality for the examination of cervical lymph nodes. However, the deficiency in the number of patients might not allow to generalise our findings to the general populations.

## INTRODUCTION

Cervical lymph nodes consist of lymphoid tissue and lymphatic drainage sites of the oral cavity and located along the lymphatic vessels in the neck [1]. The lymph nodes undergo reactive changes in response to various stimuli which contain microbial agents, chemicals, tissue damage, immune complexes and neoplasia [2]. The cervical lymph nodes are evaluated through computed tomography (CT), but it is less susceptible than ultrasonography (USG) to identify of small nodes [3]. Also lymph nodes are evaluated through magnetic resonance imaging (MRI), but calcification within lymph nodes is not readily defined with MRI [3]. Ultrasonographic examination of cervical lymph nodes is common in clinical application with high sensitivity and specificity due to noninvasive, radiation free, real-time imaging information [4].

The cervical lymph nodes grouped into seven levels using the American Joint Committee on Cancer (AJCC) classification. This classification is generally used by surgeons and oncologists but not specific for ultrasonographic examination [3]. So Hajek *et al*. [5] identified an alternative classification for ultrasonographic analysis of cervical nodes. In this classification, the nodes were grouped into eight areas by their position in the neck. However it should not be used for staging of cancers which is based on the AJCC classification [3].

Gray scale ultrasonographic nodal features which include size, shape, border sharpness, presence of an echogenic hilum aid in the differantion of normal from abnormal lymph nodes [6]. The Doppler ultrasonography is used to assess the vascularity pattern [7]. It is crucial that evaluation of cervical lymph nodes for patients with head and neck malignancies, is beneficial in defining patient prognosis, and in deciding appropriate treatment procedure [8]. Before making a precise diagnosis of pathological changes in lymph nodes, an obvious knowledge of the normal features of cervical nodes is important [3].

The size of cervical lymph nodes differs with the position in distinct regions of the neck [3]. Lymph nodes in the submandibular and upper cervical regions tend to be greater than the lymph nodes in other regions of the neck [3, 9, 10]. An inflammation in the mouth may cause the improvent of reactive changes in these lymph nodes [3, 10].

The size of lymph nodes is measured along two axis: the short axis (S) and the long axis (L) [2, 3, 10]. The shape of lymph nodes is usually evaluated by the S/L ratio. A lymph node with a S/L ratio less than 0.5 is oval in shape, however a S/L ratio greater than 0.5 seems round in shape [11]. An oval node demonstrates normality, while malignant nodes tend to be round. However, the normal submandibular nodes have usually a S/L ratio greater or equal than 0.5 [10]. The presence of an echogenic hilum is usually contemplated as an indication of benignity and it is commonly seen in larger nodes [12]. It has been reported that the frequency of echogenic hilus in normal nodes rises with age [13].

The vascular character of cervical nodes is generally classified into four types: hilar, peripheral, mixed and apparently avascular [14]. Normal and reactive lymph nodes usually seem hilar vascularity or apparently avascular whereas malignant nodes show peripheral or mixed vascularity [3, 10].

It is feasible that nodal morphology and vascular pattern of lymph nodes are affected by age, gender and body mass index (BMI). BMI is a parameter of body fat based on height and weight that applies to adults and children and is the cornerstone of the current classification for obesity [15] and cancer [16]. Higher BMI and a longer short-axis diameter may show that patients with wider body size may also have wider lymph nodes [11].

To our knowledge, only one reported study has evaluated the relation between the BMI and nodal form that was performed in Chinese population [11] and there is no reported study with Turkish population. Therefore, the aim of the presented study was to evaluate whether there was any relation between age, gender and body mass index (BMI) and nodal forms and vascular type in healthy Turkish adults subpopulation.

## MATERIALS AND METHODS

The study was carried out at the Department of Oral Diagnosis and Radiology, Marmara University Faculty of Dentistry. Twenty five subjects (12 male, 13 female) who were aged from 21 to 58 years, with no anamnesis of head and neck operation, carcinoma, tuberculosis and chronic tonsillitis participated. Confirmation for the study was acquired from the Department of Non-invasive Clinical Research Ethics Committee, Marmara University Faculty of Medicine (Project No: 092015139). The written informed consent was acquired from each subject to inform about the study’s object and procedure.

Ultrasonography was performed by two oral and maxillofacial radiologist (Ö.O. and M.D.). Body height and body weight were measured at the time of the USG examination. BMI was calculated as weight divided by the square of height (kg/m^2^). Ultrasonographic evaluations were applied using an ALOKA Prosound Alpha 6 (Hitachi Aloka Medical Systems, Tokyo, Japan) and the images were obtained with a 7.2 MHz linear array transducer. Doppler sonography was applied to assess the vascularity of lymph nodes. The submental Fig. (**1**), upper cervical Fig. (**2**) and submandibular Fig. (**3**) nodes were evaluated bilaterally by using Hajek’s classification [5].

The subjects were positioned supine on the examination couch with the neck hyperextended. The evaluation began with a horizontal scan of submental region. Then, the subject’s head was turned towards the opposite side and scanning was followed with series from the submandibular to the upper cervical region. The ultrasonographic properties such as size, shape, short axis/long axis ratio (S/L), hilum were evaluated and recorded.

The data were analysed with the IBM SPSS statistics (IBM SPSS, Turkey) 22.0. The compliance with the normal distribution of parameters was assessed with Shapiro Wilks test. In comparison, the descriptive statistical methods and quantitative data between two groups Student t test was used. The Pearson correlation analysis was used for examining the relationship between parameters.

## RESULTS

The mean age of subjects was 31.84±12.80. The ratio of short to long axis diameter (S/L) in right and left cervical nodes, submandibular nodes and submental nodes were shown in (Table **1**). The ratios of S/L in right and left cervical nodes were calculated as 0.21-0.64 mm and 0.22-0.46 mm in right and left cervical nodes, respectively. The ratios of S/L were calculated as 0.19-0.54 mm and 0.2-0.66 mm in right and left submandibular nodes, respectively. The ratios of S/L were calculated as 0.2-0.66 mm and 0.18-0.48 mm in right and left submental nodes, respectively.

The ratios of lymph nodes with avascular type were 96% for the upper cervical lymph area, 92% for the submandibular area and 96% for the submental area. The remaining lymph nodes were showed hilar vascularity.

(Table **2**) presents that there was no statistically significant difference between the subjects' ages and the ratio of short to long axis diameter (S/L) of lymph nodes in all regions (*p*>0.05).

(Table **3**) shows that among the subjects' BMI and the ratio of short to long axis diameter (S/L) of lymph nodes in all regions were not found statistically significant difference (*p*>0.05).

(Table **4**) presents that among the subjects' gender and the ratio of short to long axis diameter (S/L) of lymph nodes in all regions were not found statistically significant difference (*p*>0.05).

## DISCUSSION

Ultrasonography (USG) has many advantages involving radiation free, broadly available, simple-to-use, noninvasive, inexpensive [17]. USG is a helpful imaging technique in the evaluation of cervical lymph nodes. For a proper examination of this region it is essential that the clinician has good information of the normal anatomy of the region [18]. So ultrasound examination of soft tissue structures including cervical lymph nodes is applicable by clinicians for routine practice. The ultrasonographic appearance of normal lymph nodes differs from the abnormal nodes. Sonographic characteres that may be assessed and help in distinguishing normal from pathologic lymph nodes include the following: node size, shape, border sharpness, presence of an echogenic hilum and vascularity [19]. A normal lymph node should be oval, with a hilus, sharp margins and avascular or hilar vascularity [20].

Most prior works of ultrasonographic characters of nodes in the cervical region have centered on pathologic lymph nodes [21-23] while the normal anatomy of cervical lymph nodes and their association between age, gender, BMI was investigated in the study. van den Brekel *et al.* [21] examined 55 patients with head and neck cancer and lymph nodes’ location, number, diameter, and the quantity of necrosis and fatty metaplasia were recorded. They reported that the minimum diameter in the axial plane was the most proper size measure for forecasting lymph node metastasis and a minimum axial diameter of 10 mm was defined to be the most efficient size criterion.

Ying *et al.* [13] reported that 133 healthy subjects (67 men and 66 women) had performed ultrasonographic investigations of the neck and 1299 lymph nodes were found. The nodes were evaluated for their size, shape (short-to-long-axis ratio), border sharpness and for the entity of an echogenic hilum. The samples were classified by age (20-29, 30-39, 40-49, and > or = 50 years) and subclassified by gender. They observed that nodal size has no significantly difference between gender and age groups. Alike, this study has also found no stastically significant difference between gender and age groups and nodal size (p>0.05). Osanai *et al*. [11] similarly with our results, no significant relation was shown between age and the S/L ratio. On the contrary Marchal *et al*. [24] hypothesized that normal nodes in younger subjects tend to be smaller than those in older subjects. This distinction may be associated the raised fatty deposition in nodes in the elders [24].

Nodal shape and size are also useful criteria for distinguishing normal and pathologic lymph nodes [25, 26]. Tohnosu *et al*. [25] reported that the S/L ratio of lymph node exceeding 0.5 demonstrated a much higher rate of metastasis than S/L under 0.5. The authors identified 126 lymph nodes in 58 patients with esophageal cancer. They observed the mean cancer content in the metastatic lymph nodes with S/L under 0.5 and over 0.5 was 26.0% and 59.1%, respectively. Most the S/L ratios of lymph nodes were over 0.5. Contrary, in presented study, most the S/L ratios of lymph nodes were observed under 0.5 with oval shape. Ahuja and Ying [27] reported that metastatic lymph nodes were round shaped with S/L over 0.5 while benign nodes were oval shaped with S/L under 0.5. Also they verified that the common ultrasonographic features of different reasons of cervical lymphadenopathy explored with comparing the normal anatomy of cervical lymph nodes.

In this study it is shown that no statistically significant difference was found between gender and the ratio of short to long axis diameter (S/L) of lymph nodes in all regions (*p*>0.05). Contrary, Osanai *et al.* [11] reported that the S/L of female was significantly larger than that of male.

To our knowledge the present study was the first study evaluating the relation of age, gender and BMI with vascular pattern and nodal morphology in Turkish people. Osanai *et al*. [11] found that a higher BMI was significantly related with S/L ratio in all regions, whereas our results observed no significant difference between BMI and nodal size (*p*>0.05). Kawamoto *et al*. [28] assessed the relation between BMI and high sensitivity C-reactive protein (CRP) concentrations and found that adiposity increases low-grade systemic inflammation. C-reactive protein (CRP) is a crucial inflammation sensitive plasma protein in humans [29]. Similarly in the other study it has been reported that higher BMI is related with higher CRP concentrations [29]. Thus low-grade systemic inflammation may be a possible process relation gender and BMI with nodal form [11]. Osanai *et al*. [11] suggested that gender and BMI should be considered when lymphadenopathy is determined as well as the presence of metastatic lymph nodes.

Normal lymph nodes frequently have an echogenic hilum, while malign nodes generally do not [28, 29]. Toriyabe *et al.* [30] reported that benign nodes had internal echo structure with eccentric hyperechoic. At the same time Vassallo [31] reported that the central hilus was the sonographic criteria for differentiation of benign from malignant lymph nodes. In this study, all the examined lymph nodes were a central or eccentric hilus, consistent with the previous studies.

Normal cervical lymph nodes have apparently avascular or hilar vascularity [10]. Ying and Ahuja [10] reported that the grade of vascularity of submental and submandibular lymph nodes is significantly higher than the other regions. The most of lymph nodes showed avascular pattern in this study. The ratio of lymph nodes with a vascular type was 96% for the upper cervical lymph area, 92% for the submandibular area and 96% for the submental area. However, Osanai [11] showed a hilar or avascular type were 35% for the upper cervical area, 20% for the submandibular area and 21% for the submental area. Also hilar vascularity was observed as the most common pattern. This difference may be associated with inadequate number of patients.

On the other hand, a limitation should be recognized in the study. The deficiency in the number of patients might not allow to generalise our findings to the general population.

## CONCLUSION

In conclusion, normal cervical lymph nodes are oval, with an unsharp border and an echogenic hilum but no relation was found between lymph nodes and the age, gender and BMI.

## Figures and Tables

**Fig. (1):**
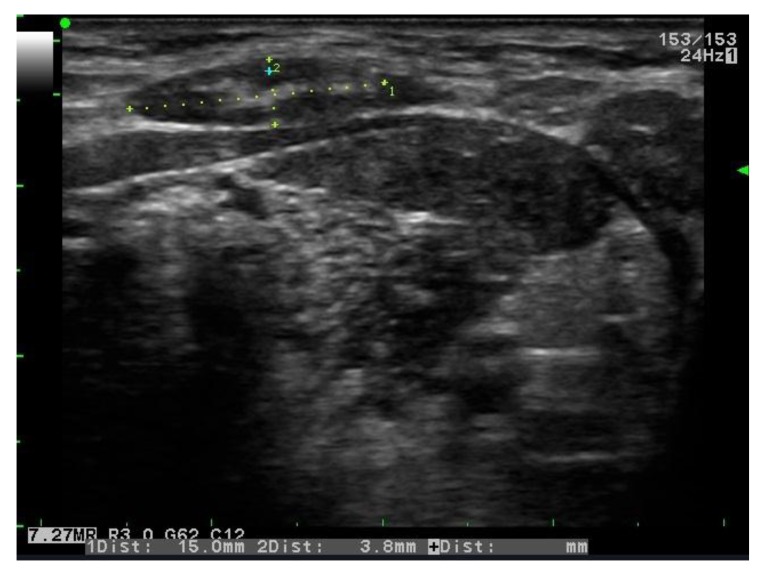
Gray scale US: right submental lymph node with oval shape, echogenic hilus and S/L<0.5.

**Fig. (2):**
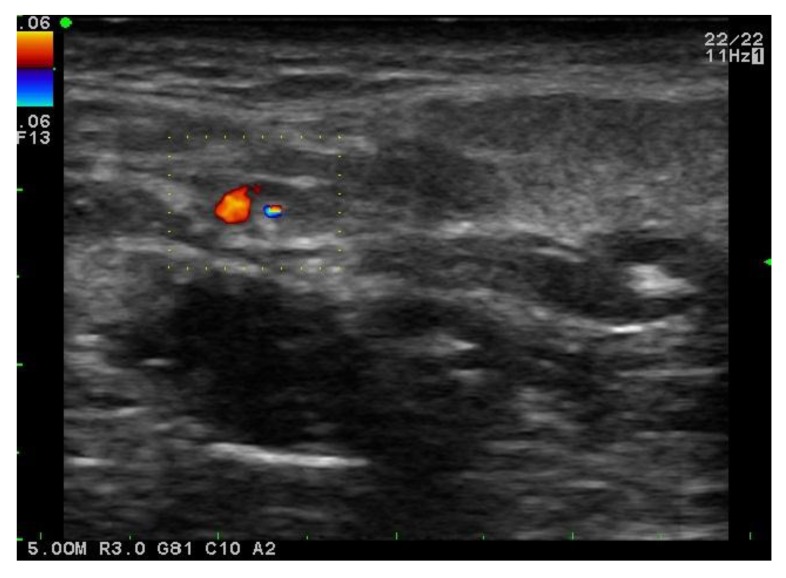
Doppler US: right upper cervical lymph node with hilar vascularity.

**Fig. (3):**
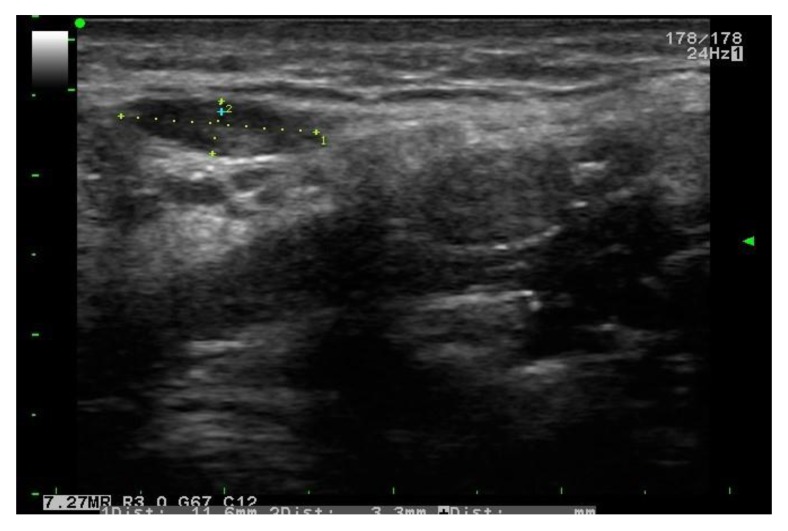
Gray scale US: right submandibular lymph node with oval shape, echogenic hilus and S/L<0.5.

**Table 1:** The distribution of size of lymph nodes.

	**Right**		**Left**	
	**Min-Max**	**Mean±SD**	**Min-Max**	**Mean±SD**
**Cervical region**				
**Short-axis diameter (mm)**	2-5	3.56±0.73	2.7-5.5	3.84±0.75
**Long-axis diameter (mm)**	5.9-18	11.11±3.36	7.1-16.9	11.8±2.67
**S/L**	0.21-0.64	0.34±0.11	0.22-0.46	0.34±0.07
**Submandibular region**				
**Short-axis diameter (mm)**	1.8-5,8	3.87±1.01	2.6-7.7	4.24±1.07
**Long-axis diameter (mm)**	5.7-23.3	12.84±4.4	7.6-16	11.94±1.95
**S/L**	0.19-0.54	0.33±0.11	0.2-0.66	0.36±0.11
**Submental region**				
**Short-axis diameter (mm)**	2.4-8.8	4.65±1.29	2.3-8	4.54±1.28
**Long-axis diameter (mm)**	7.4-25.2	15.19±4.62	9.6-29	16.18±4.72
**S/L**	0.2-0.66	0.32±0.1	0.18-0.48	0.29±0.07

**Table 2:** Correlation of lymph nodes by age.

	**Age**			
	**Right**		**Left**	
	***r***	***p***	***r***	***p***
**Cervical region**				
**Short-axis diameter (mm)**	0.019	0.927	0.408	0.043
**Long-axis diameter (mm)**	-0.155	0.459	0.236	0.256
**S/L**	0.134	0.524	0.124	0.554
**Submandibular region**				
**Short-axis diameter (mm)**	-0.044	0.834	0.215	0.303
**Long-axis diameter (mm)**	-0.071	0.737	-0.115	0.585
**S/L**	0.107	0.609	0.208	0.318
**Submental region**				
**Short-axis diameter (mm)**	0.122	0.561	0.016	0.941
**Long-axis diameter (mm)**	-0.010	0.962	0.070	0.741
**S/L**	0.141	0.503	-0.075	0.722

Pearson Correlation Test *p*<0.05

**Table 3:** Correlation of lymph nodes by BMI.

	**BMl**			
	**Right**		**Left**	
	***r***	***p***	***r***	***p***
**Cervical region**				
**Short-axis diameter (mm)**	0.071	0.735	0.335	0.102
**Long-axis diameter (mm)**	0.073	0.728	0.153	0.465
**S/L**	-0.066	0.756	0.124	0.553
**Submandibular region**				
**Short-axis diameter (mm)**	0.081	0.701	0.260	0.210
**Long-axis diameter (mm)**	0.026	0.902	-0.038	0.857
**S/L**	-0.006	0.978	0.210	0.313
**Submental region**				
**Short-axis diameter (mm)**	0.199	0.341	0.195	0.350
**Long-axis diameter (mm)**	0.192	0.358	0.141	0.502
**S/L**	-0.098	0.642	0.026	0.903

Pearson Correlation Test *p*<0.05

**Table 4:** Correlation of lymph nodes by gender.

	**Right**		***p***	**Left**		***p***
	**Female**	**Male**		**Female**	**Male**	
	**Mean±SD**	**Mean±SD**		**Mean±SD**	**Mean±SD**	
**Cervical region**						
**Short-axis diameter (mm)**	3.59±0.78	3.53±0.7	**0.823**	3.7±0.75	3.99±0.76	**0.345**
**Long-axis diameter (mm)**	11.35±4.04	10.85±2.6	**0.717**	11.75±2.52	11.85±2.95	**0.931**
**S/L**	0.34±0.12	0.34±0.1	**0.926**	0.33±0.08	0.35±0.07	**0.403**
**Submandibular region**						
**Short-axis diameter (mm)**	3.82±1.02	3.92±1.05	**0.823**	3.85±0.59	4.65±1.34	**0.063**
**Long-axis diameter (mm)**	12.45±3.55	13.26±5.3	**0.654**	12.18±2.32	11.68±1.51	**0.533**
**S/L**	0.33±0.12	0.32±0.1	**0.836**	0.32±0.07	0.40±0.12	**0.055**
**Submental region**						
**Short-axis diameter (mm)**	4.29±0.85	5.04±1.6	**0.152**	4.27±0.73	4.83±1.68	**0.301**
**Long-axis diameter (mm)**	13.85±3.15	16.64±5.6	**0.147**	15.45±2.65	16.98±6.29	**0.445**
**S/L**	0.33±0.12	0.31±0.07	**0.665**	0.28±0.07	0.29±0.06	**0.741**

Student t Test *p*<0.05
